# Comparative genomics of a novel clade shed light on the evolution of the genus *Erysipelothrix* and characterise an emerging species

**DOI:** 10.1038/s41598-021-82959-x

**Published:** 2021-02-09

**Authors:** Ana Laura Grazziotin, Newton M. Vidal, Patricia Giovana Hoepers, Thais F. M. Reis, Dany Mesa, Luiz Felipe Caron, Max Ingberman, Breno C. B. Beirão, João Paulo Zuffo, Belchiolina Beatriz Fonseca

**Affiliations:** 1 Programa de Pós-Graduação em Ciências Veterinárias, Faculdade de Medicina Veterinária, Universidade Federal de Uberlândia, Rua Ceará, 1084, Bloco 2D, Sala 54, Umuarama, Uberlândia, MG CEP: 38405-240 Brasil; 2 Programa de Pós-Graduação em Biodiversidade Animal, Departamento de Evolução e Ecologia, Universidade Federal de Santa Maria, Avenida Roraima, 1000, Prédio 17, Sala 1140-D, Cidade Universitária, Bairro Camobi, Santa Maria, RS CEP: 97105-900 Brasil; 3 Departmento de Bioquímica e Biologia Molecular, Universidade Federal do Paraná, Curitiba, PR Brasil; 4 Departamento de Patologia Básica, Setor de Ciências Biológicas, Universidade Federal do Paraná, Curitiba, PR Brasil; 5 Imunova Análises Biológicas, Curitiba, PR Brasil; 6 Centro de Diagnóstico de Microbiologia Animal, Universidade do Estado de Santa Catarina, Florianópolis, SC Brasil

**Keywords:** Microbial genetics, Classification and taxonomy

## Abstract

*Erysipelothrix* sp. isolates obtained from a deadly outbreak in farmed turkeys were sequenced and compared to representatives of the genus. Phylogenetic trees—supported by digital DNA:DNA hybridization and Average Nucleotide Identity—revealed a novel monophyletic clade comprising isolates from pigs, turkeys, and fish, including isolates previously described as *E.* sp. Strain 2. Genes coding for the SpaC protein, typically found in *E.* sp. Strain 2, were detected in all isolates of the clade. Therefore, we confirm *E.* sp. Strain 2 represents a unique species, that despite its official name “*Erysipelothrix piscisicarius*” (meaning a killer of fish), may be isolated from a broad host range. Core genome analysis showed that the pathogenic species of this genus, *E. rhusiopathiae* and the clade *E.* sp. Strain 2, are enriched in core functionalities related to nutrient uptake and transport, but not necessarily homologous pathways. For instance, whereas the aerobic DctA transporter may uptake C_4_-dicarboxylates in both species, the anaerobic DcuC transporter is exclusive of the *E.* sp. Strain 2. Remarkably, the pan-genome analysis uncovered that genes related to transport and metabolism, recombination and repair, translation and transcription in the fish isolate, within the novel clade, have undergone a genomic reduction through pseudogenization. This reflects distinct selective pressures shaping the genome of species and strains within the genus *Erysipelothrix* while adapting to their respective niches.

## Introduction

Bacterial comparative genomics analyses have brought to light unprecedented aspects of bacterial physiology, diversity and evolution^1^. Uncovering the genomic repertoire of bacterial organisms has also revealed an extensive intraspecific diversity^2^. Therefore, whole-genome sequencing (WGS) has become a powerful tool not only for detecting genetic features and specific adaptations but also for taxonomy, assisting in species delineation^3^. Phylogenomics and whole-sequence alignment-based metrics, such as digital DNA:DNA hybridization (dDDH) and Average Nucleotide Identity (ANI), have been widely used and supported the identification of novel species and reclassification of known taxons^3–6^. In addition, components of the genomic repertoire (core, pan-genome and unique genes) may provide supporting evidence for bacterial characterization and species definition. For instance, the presence of species-specific core genes, lineage-specific expansions or gene losses make up a bacterial genomic identity and reflect adaptive strategies.

A number of complete bacterial genomes of the genus *Erysipelothrix* (family Erysipelotrichaceae*,* phylum Firmicutes) have been made available in the past years. The first genome, *E. rhusiopathiae* strain Fujisawa, was released in 2011^7^ and showed that the organism lacks many biosynthetic pathways, which was also observed in *E. rhusiopathiae* SY1027^8^, indicating a reductive genome evolution. Since then many more genomes of the same and other species have been published, providing an opportunity to assess their genetic variations, functional traits and reconstruct ancestral trajectories. An in depth analysis of *E. rhusiopathiae* genomes from a worldwide population showed that the species comprises three distinct clades with weak association to host or geographic origin^9^. Conversely, a WGS study of *E. rhusiopathiae* from a Japanese swine outbreak showed the strains were closely related with few SNPs (single nucleotide polymorphisms) among them and four main lineages were responsible for the acute disease^10^. Most studies, however, have focused on characterizing *Erysipelothrix* species or strains based mainly on serology, spa proteins, and genotype, based on molecular techniques such as pulsed-field gel electrophoresis^11–13^. The phylogenetic reconstruction, phenotypic characterization and pathogenic potential of the genus *Erysipelothrix* were covered in a study of the family *Erisipelotrichaceae*^14^, which redefined two genera within the family. However, no comprehensive comparative genomic analysis of the genus *Erysipelothrix* has been carried out to date. Moreover, *E. rhusiopathiae* has been vastly studied whereas studies focusing on other *Erysipelothrix* species are very scarce, limiting our understanding of ecological aspects, diversity, genetic traits and evolutionary scale.

Currently, the *Erysipelothrix* genus comprises five named species, *E. rhusiopathiae*^15^, *E. tonsillarum*^16^, *E. inopinata*^17^, *E. larvae*^18^ and *E. piscisicarius*^19^. *E. rhusiopathiae* is the best characterized species, responsible for a spectrum of diseases in humans and wild and domestic animals^20^. *E. tonsillarum* has been isolated from healthy swine tonsils^16^ and also from dogs with endocarditis^21,22^. *E. inopinata* was isolated from a broth culture^17^ and *E. larvae* seems to be a commensal species of a beetle gut^18^. In addition, other potential novel species of the genus have been indicated, such as *E.* sp. Strain 1, *E.* sp. Strain 2 and *E.* sp. Strain 3^11,23,24^. The first two, *E.* sp. Strain 1 and *E.* sp. Strain 2, were isolated from pigs and previously identified as *E. rhusiopathiae* strain Pécs 56 (serovar 13) and strain 715 (serovar 18), respectively^23^ until they were shown to be very dissimilar from either *E. rhusiopathiae* and *E. tonsillarum* type strains as well as from each other based on DDH experiments, suggesting they represented novel species^23^. A third group of distinct isolates, *E.* sp. Strain 3, was also identified^24^. *E.* sp. Strain 1 and Strain 3 have been poorly characterized to date. In contrast, *E.* sp. Strain 2 (type strain 715) has been studied and at least three serovars (9, 10 and 18) are associated with this strain, which were found to be pathogenic in mice and pigs^24^; it carries a molecular variant (*spaC*) of the surface protective antigen protein^25^ and; it is phylogenetic distinct from *E. rhusiopathiae* and *E. tonsillarum*^9^. Recently, deadly outbreaks in farmed fish and turkeys were associated with *E.* sp. Strain 2^26,27^. Although the ANI analysis between the fish isolate genome (isolate 15TAL0474) and the swine isolate genome (type strain 715) showed they are highly similar (above 99% similarity), slight but consistent differences based on a MLSA tree were observed between the two isolates and thus, authors proposed the fish isolate as a novel species with the name *E. piscisicarius*^19^. Given that *E.* sp. Strain 2-related isolates have been shown to cause lesions in pigs and mice^24^ and death of farmed fish^19,26^ and turkeys^27^, this is likely to be an economically important pathogen in animal production. Nevertheless, limited information is available regarding its biology and, since only recently a representative genome has become available^19^, the understanding of its population diversity and genome evolution is still scarce.

In this study, we sequenced isolates from the turkey outbreak^27^ and compared them to the representative species of the genus *Erysipelothrix*. We hypothesized that the emergent pathogenic *Erysipelothrix* isolates from recent outbreaks in turkey and fish belong to a single genomospecies (a species that can be differentiated from other species based on genomic methods), which is apart from the other well characterized *Erysipelothrix* species. Therefore, we investigated the presence of Spa proteins and the phylogenetic relationship amongst all current species of the genus using publicly available genomes. Whole genome-based similarity metrics (dDDH and ANI) were also performed to confirm the taxonomic relationship. After, the genomic repertoires within and among species were assessed, focusing on the novel emergent species, in order to identify shared and specific genetic features related to the species diversity, genome evolution and specific adaptations within the genus.

## Results and discussion

### The 16S rRNA phylogenetic tree is not suitable for delineating *Erysipelothrix* species

**Figure 1:**
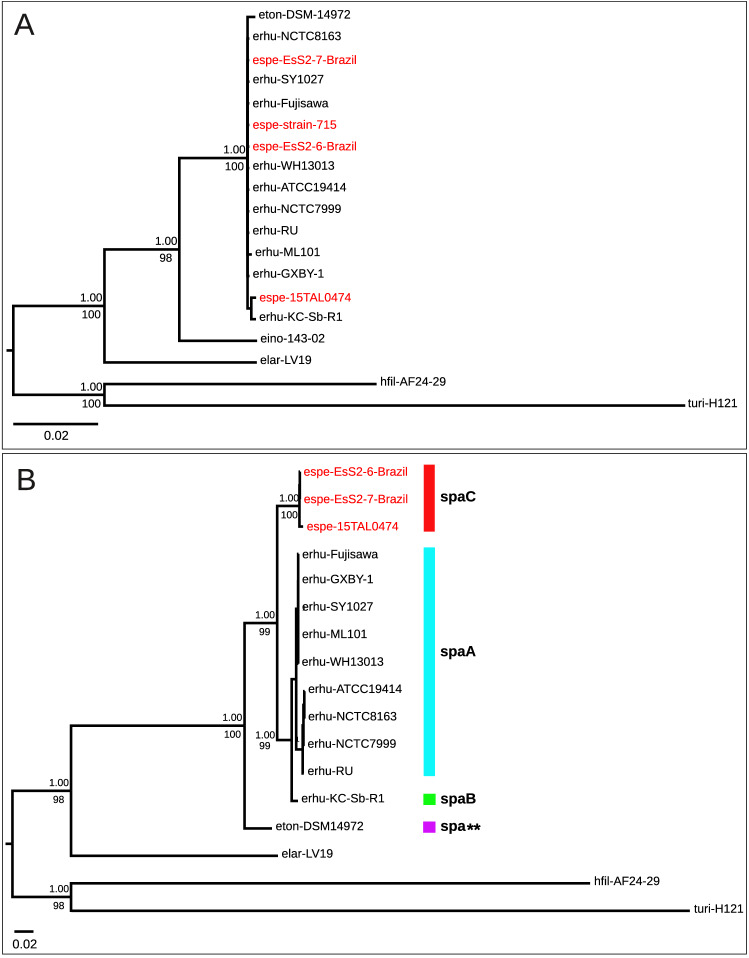
Phylogenetic reconstruction of *Erysipelothrix* genus using single-genes. Legend: (**A**) Bayesian phylogenetic tree based on 16S rRNA gene under the model GTR+I+G. (**B**) Bayesian phylogenetic tree based on rpoB nucleotide sequence under the model GTR+I+G. Posterior probability values of support obtained in Bayesian Analysis (BA) are shown above nodes. Rapid bootstrap values obtained in Maximum Likelihood (ML) analysis are shown below nodes. Species were indicated as follows: *E.* sp. Strain 2 isolates (espe-EsS2-6-Brazil, espe-EsS2-7-Brazil, espe-15TAL0474, espe-strain-715); *Erysipelothrix rhusiopathiae* isolates (erhu-ATCC19414, erhu-KC-Sb-R1, erhu-NCTC8163, erhu-NCTC7999, erhu-SY1027, erhu-GXBY-1, erhu-WH13013, erhu-Fujisawa, erhu-ML101, erhu-RU); *Erysipelothrix inopinata* strain 143-02 (eino-143-02); *E. tonsillarum* DSM 14972 (eton-DSM14972); *E. larvae* LV19 (elar-LV19); *Holdemania filiformis* AF24-29 (hfil-AF24-29); *Turicibacter* sp. H121 (turi-H121). Species hfil-AF24-29 and turi-H121 were used as outgroups. *E.* sp. Strain 2 isolates are shown in red.

Full length 16S rRNA sequences were retrieved from available genomes (Supplementary Table S1). Sequences from *E. inopinata* and *E.* sp. Strain 2 (type strain 715) were retrieved from NCBI Nucleotide since no genome sequences were publicly available. The 16S rRNA gene was used since it has been a long-standing primary choice for bacterial diagnosis and identification. Based on the 16S rRNA gene tree, *Erysipelothrix* species formed three distinct clades (Fig. 1A). *E. larvae* was shown as the most ancestral species of the genus *Erysipelothrix*, followed by *E. inopinata*, each one was placed in a highly supported single branch on the tree. However, the remaining isolates belonging to *E. tonsillarum*, *E. rhusiopathiae* and *E.* sp. Strain 2 (isolates 15TAL0474, EsS2-6-Brazil, EsS2-7-Brazil and type strain 715) were clustered all together, supported by pairwise sequence similarities above 99% (Supplementary Table S2), which is higher than the standard threshold value (97%) used as species boundaries^28^. Therefore, 16S rRNA sequences are not recommended to distinguish among *Erysipelothrix* species.

Thus, we used the housekeeping gene rpoB (beta subunit of RNA polymerase) to check the phylogenetic relatedness (Fig. 1B). The rpoB gene has been suggested as an alternative for the 16S rRNA gene due to its universality, ancient origin and sufficient number of sequence variation to discriminate bacterial species^29^ and, therefore, it has been applied for bacterial identification of clinical isolates^30,31^. The rpoB gene tree showed a clear distinction of *Erysipelothrix* species (Fig. 1B). Remarkably, the three *E.* sp. Strain 2-related isolates (15TAL0474, EsS2-6-Brazil and EsS2-7-Brazil) formed a highly supported monophyletic group, indicating that these isolates might represent a new taxon. Accordingly, the three isolates showed 99.61–99.98% identity within the group (Supplementary Table S2), which is above the proposed threshold for a new bacterial species (97.7%)^32,33^ and subspecies (98.2%) delineation^29^, indicating that these isolates might belong to the same species. *E. inopinata* and *E.* sp. Strain 2 (type strain 715) were not included in this and further analysis since no rpoB gene sequence nor their genome sequences were publicly available during the time this work was performed and manuscript was written.

### The SpaC protein sequence is present in all *E.* sp. Strain 2-related isolates and a novel Spa variant is found in *E. tonsillarum*

We investigated the presence of the surface protective antigen protein (Spa) sequence since the presence of the SpaC variant has been suggested to distinguish *E.* sp. Strain 2 from other *Erysipelothrix* spp.^25^. The typical SpaC was found in all *E*. sp. Strain 2-related isolates whereas SpaA and SpaB were found in *E. rhusiopathiae* (Fig. 1B), as expected^25,34^. No Spa sequence was detected in *E. larvae* but surprisingly, a Spa protein sequence was found in *E. tonsillarum* (Supplementary Fig. S1A). The novel Spa protein sequence is distantly related to the other Spa types showing the lowest identities (43.8% Spa A, 41.1% SpaB and 37.9% SpaC) amongst them (Supplementary Fig. S1B). Previous studies of *spa* gene detection based on PCR have not found a *spa* sequence in *E. tonsillarum*^12,25,35,36^ and only a single work reported the detection of *spaA* and *spaB* in *E. tonsillarum* by PCR^26^, but the fragments were not sequenced. Experimental or genomic studies assessing the prevalence of Spa protein in other *E. tonsillarum* isolates may clarify the extension of its presence in the species.

### Multilocus sequence analysis (MLSA) and phylogenomics reconstructions show a novel species within the *Erysipelothrix* genus

**Figure 2:**
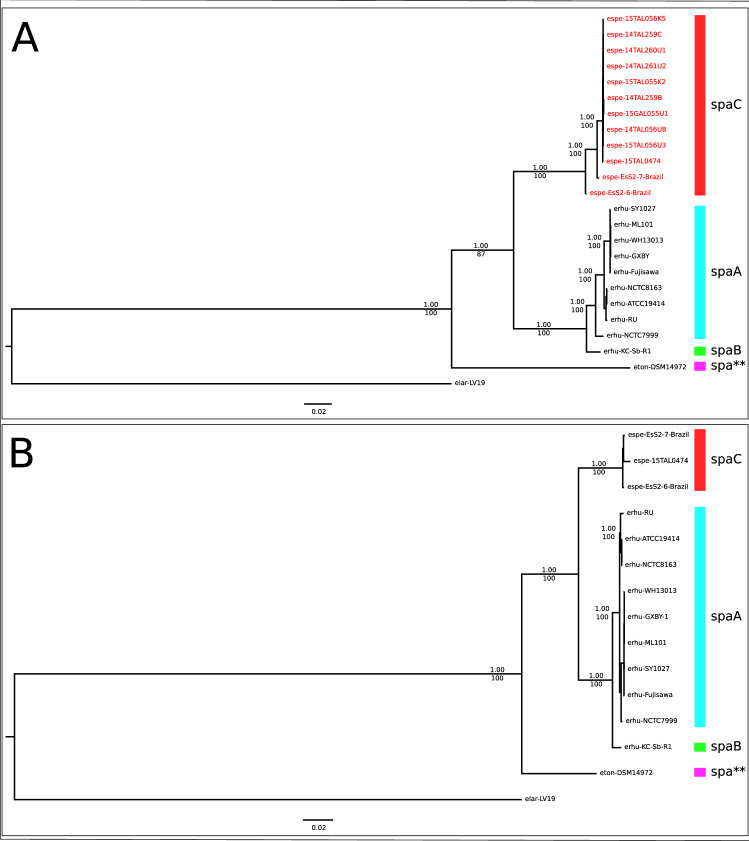
Phylogenetic reconstruction of *Erysipelothrix* genus using Multilocus Sequence analysis (MLSA) and phylogenomic analysis. Legend: (**A**) MLSA Bayesian phylogenetic tree based on the partitioned supermatrix of seven genes (galK, gpsA, ldhA, prsA, pta, purA and recA). (**B**) Phylogenomic Bayesian phylogenetic tree based on the partitioned supermatrix of 506 single-copy core-genome orthologous proteins. Posterior probability values of support obtained in Bayesian Analysis (BA) are shown above nodes. Rapid bootstrap values obtained in Maximum Likelihood (ML) analysis are shown below nodes. Species were indicated as described Supplementary Table S1.

Next, we used multilocus sequence approaches to verify the species relatedness within the *Erysipelothrix* genus. In recent years, MLSA and phylogenomics have been widely used to discriminate bacterial species and strains^3,37,38^ due to their higher resolution compared to single-locus approaches. The MLSA tree (Fig. 2A) is based on seven slowly evolving gene sequences (galK, gpsA, ldhA, prsA, pta, purA and recA) previously proposed for multilocus sequence typing of *E. rhusiopathiae*^13^. In addition to our sequenced genomes and publicly available genomes from various hosts, the MLSA phylogeny included gene sequences from nine other fish isolates (*E.* sp. Strain 2-related isolates), whose genome sequences, although reported, were not made publicly available^19^. The phylogenomic tree (Fig. 2B) is based on the alignment of 506 single-copy orthologous proteins for the *Erysipelothrix* genus. The MLSA and the phylogenomic trees are topologically similar, showing four well-supported clades. *E. larvae* and *E. tonsillarum* form the deepest branches of the trees whereas the two most derived clades split *E. rhusiopathiae* from the newly sequenced *E.* sp. Strain 2-related isolates. The latter group also included all 10 isolates collected from fish during a disease outbreak in the United States^19,26^ by MLSA. The consistent monophyletic nature of *E.* sp. Strain 2-related isolates based on three distinct phylogenetic approaches is the main criterion for defining a novel taxon^39^.

### Whole-genome alignment analyses (dDDH and ANI) confirm the phylogenomic relatedness

**Figure 3:**
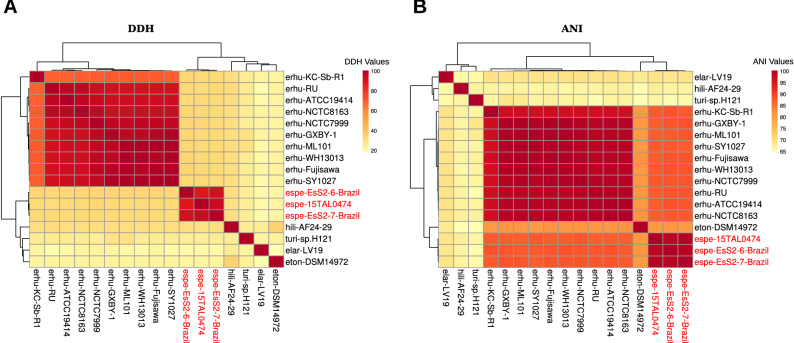
Heatmap of whole-genome sequence pairwise comparisons between species of the genus *Erysipelothrix* and two outgroups. Legend: (**A**) Heatmap of the digital DNA:DNA hybridizations (dDDH). (**B**) Heatmap of the Average Nucleotide Identity (ANI). The percentages are provided in Supplementary Table S3. Species mentioned in the figures are described Supplementary Table S1.

To confirm the species relatedness inferred from the phylogenetic trees and ensure an accurate assignment at the species level, the pairwise nucleotide-level comparisons (dDDH and ANI) were calculated for 15 genomes of genus *Erysipelothrix* and closely related genera (Fig. 3A,B). The established same-species delineation thresholds are 70% for dDDH^40,41^ and 95% for ANI^42^ values. The dDDH and ANI values between all pairs of *E.* sp. Strain 2-related genomes and *E. rhusiopathiae* genomes were below both thresholds (dDDH 31.5–33% and ANI 86.76–87.83%) (Supplementary Table S3), confirming that they represent distinct species at the genome level. Of note, amongst *E.* sp. Strain 2-related genomes all metrics were above the threshold (dDDH 87.1–92.9% and ANI 98.51–99.14%) (Supplementary Table S3), providing further evidence that these isolates comprise a genomospecies, as supported by the monophyletic clade in rpoB tree, MLSA and phylogenomics.

The two combined approaches—phylogenomics and whole-genome nucleotide metrics—demonstrated that isolates related to *E*. sp. Strain 2 belong to the same species. The type strain 715 was previously isolated from a swine spleen and distinguished from *E. rhusiopathiae* based on a wet lab DDH approach^23^. At that time, authors suggested that isolate 715 could represent a novel species but to date, no study has comprehensively characterized such isolate. *E. s*p. 15TAL0474, isolated from fish, has been recently sequenced and compared to the pig isolate (type strain 715) by dDDH (90.8%) and ANI (99.01%)^19^, which supported that these strains would belong to the same species. However, due to a slight but consistent variation in MLSA pattern between the pig and the fish isolates, authors considered the fish isolate a novel species, which was named *E. piscisicarius*^19^. Intraspecific variation is commonly observed within many species^9,43^ and the genotypic diversity within *E. rhusiopathiae* has been already demonstrated^9^. For instance, the variation found between the pig and the fish genomes^19^ is no greater than that found within *E. rhusiopathiae*, i.e., between the Clade 1 (more distinct one) and the other clades of *E. rhusiopathiae*^9^ (Fig. 3AB; Supplementary Figure S2). The International Code of Nomenclature of Prokaryotes^44^ recommends that when choosing a species name (Recommendation 12c), isolates deemed conspecific should retain the species epithet provided on List of Prokaryotic names with Standing in Nomenclature. *E.* sp. Strain 2 has been isolated from a broad diversity of hosts, firstly from a pig (type strain 715)^23^, and then from fish (isolate 15TAL0474)^19^ and birds (isolates EsS2-6-Brazil and EsS2-7-Brazil)^27^. Nevertheless, though the new species represents a pathogen of multiple distinct hosts (similarly to what is observed for *E. rhusiopathiae*), and the name *E. piscisicarius* (meaning a killer of fish) does not represent the bacterium's full host spectrum, as the first taxonomically characterized and validated name for the species^19^, “*Erysipelothrix piscisicarius*” should be considered the official species name for *E. *sp. Strain 2. Given that the new species represents a pathogen of multiple distinct hosts (similarly to what is observed for *E. rhusiopathiae*) and that the name *E. piscisicarius* (meaning a killer of fish) does not represent the bacterium's full host spectrum, a more generic, unbiased name would be suitable. We suggest “*Erysipelothrix takahashiae*” after Toshio Takahashi who first discovered isolates of this clade and suggested it could represent a novel species^23^.

### The core genome of pathogenic species is overrepresented by metabolic genes

We found 917 protein families in the core genome of *E. rhusiopathiae* and *E.* sp. Strain 2 and a total of 2006 families comprising the pan-genome of both species. The core genome, as expected, is enriched (*p* < 0.05) in protein families related to the basic cellular machinery, such as “Translation, ribosomal structure and biogenesis” (Cluster of Orthologous Groups—COG category J), “Metabolism and transport of amino acids” (COG category E), “Metabolism and transport of lipids” (COG category I), and “Metabolism and transport of inorganic ions” (COG category P) (Supplementary Fig. S3; Supplementary Table S4). For some isolates functional enrichment was not statistically significant, but still their core genomes clearly showed higher proportion of genes in such categories compared to the accessory genome (Supplementary Fig. S4; Supplementary Table S5), indicating that pathways related to the metabolism of amino acids, lipids and inorganic ions play an important role for the group as a whole. Accordingly, these COG categories have been found to show a considerable number of regulated genes in *E. rhusiopathiae* HX130709 grown in rich medium^45^. After checking the list of regulated genes^45^, we found that most of the regulated genes present in COG E (68.1%), COG P (63.3%), and COG I (80%) in *E. rhusiopathiae* HX130709 belong to the core genes of *E. rhusiopathiae* and *E.* sp. Strain 2. Considering that *E. rhusiopathiae* was grown in a nutrient-rich and stress-free condition^45^, it is expected that most recruited genes are related to cell maintenance. Genes belonging to the core-genome enriched categories maintain the basic cellular machinery, the central metabolism, and mediate transport processes into and out of the cell, which means that shared genes in these categories are needed for cell growth and survival.

### Distinct core strategies of nutrient uptake and energy metabolism between *E. rhusiopathiae* and *E.* sp. Strain 2

When analysing the two species separately, 1,109 and 1,244 protein families comprised the core genome of *E. rhusiopathiae* and *E.* sp. Strain 2, respectively. The core genome represented on average 70.69% of the total coding sequences in *E. rhusiopathiae* and 82.40% for *E.* sp. Strain 2 isolates. Differences were found between the two core genomes and we highlight two protein families related to nutrient uptake and energetic metabolism.

C_4_-dicarboxylate transporters are secondary carriers for the uptake, exchange or efflux of C_4_-dicarboxylates (fumarate, succinate, aspartate and malate) from the Krebs cycle, which are relevant to the bacterial energetic metabolism when sugars are not available^46^. The DctA family of C_4_-dicarboxylate carriers (COG1301) was found in all studied *Erysipelothrix* species (*E. rhusiopathiae*, *E. tonsillarum*, *E. larvae* and *E.* sp. Strain 2), making up the core genetic repertoire of the genus (Supplementary Table S6). In contrast, the DcuC protein family C_4_-dicarboxylate transporter (COG3069) is a core protein in *E.* sp. Strain 2, which is absent in all *E. rhusiopathiae* isolates (Supplementary Table S6). Similar to *E.* sp. Strain 2, the bacterial pathogen *Campylobacter jejuni* carries both C_4_-dicarboxylate transporter genes (*dctA* and *dcuC*)^47^. DctA was the only C_4_-dicarboxylate carrier required by *C. jejuni* to grow based on dicarboxylate-carbon sources at high oxygen levels^47^ whereas under anaerobic conditions, DcuC was upregulated in the pathogen^48^. The *dcuC* gene might be induced in *E.* sp. Strain 2, similarly to other bacteria^46,48^, allowing them to transport aspartate and fumarate under oxygen-limited conditions^49,50^. Although *E. rhusiopathiae* isolates do not share an orthologous *dcuC* gene with *E.* sp. Strain 2 and apparently, they would not be able to perform C_4_-dicarboxylate transport under anaerobic condition by this route, we cannot disregard that the function might be played by a non-orthologous gene. Gene knockout mutant and transcriptome experiments of *Erysipelothrix* isolates based on dicarboxylate-carbon sources under aerobic and anaerobic conditions would help to understand the preferential metabolic strategies employed by these organisms and whether *E. rhusiopathiae* strains carry any alternative anaerobic route for dicarboxylate uptake.

The phosphoenolpyruvate (PEP)-dependent sugar phosphotransferase system (PTS) is the major carbohydrate (glucose, glucitol, mannose and ascorbate) transport system in bacteria. The PTS superfamilies comprise two cytoplasmic phosphotransferases (HPr and enzyme I—EI) and a sugar-specific permease complex (enzyme II—EII). Genes coding for HPr and EI were found in the core genome of *E. rhusiopathiae*, as well as in the other species (*E. larvae*, *E. tonsillarum* and *E*. sp. Strain 2) since their products are used to phosphorylate enzymes of all PTS superfamilies. Genes of the anaerobic L-ascorbate degradation pathway (from L-ascorbate to D-xylulose-5P (Ko00053)) belong to the operon *ulaABCDEF*^51^ and are regulated by operon *ulaGR*^52^. The anaerobic l-ascorbate degradation pathway is complete in all *E. rhusiopathiae* isolates, but two (Supplementary Fig. S5). Gene *ulaD* was missing in *E. rhusiopathiae* strain RU whereas *ulaD* and *ulaF* were missing in strain SY1027. These missing genes would be part of the core genome, however, they were considered pseudogenes due to multiple frameshift mutations. Many bacteria have been reported to ferment l-ascorbate under anaerobic conditions^51,53^ and this route may provide energy supply for survival when other sources are limited in natural environments for *E. rhusiopathiae*. In contrast, genes of the anaerobic pathway for l-ascorbate degradation were not found in *E.* sp. Strain 2. Similarly, typical l-ascorbate-related genes have not been found in *Ralstonia eutropha* genome, although the species is capable of using l-ascorbate as a sole source of carbon, which is performed via a novel catabolic pathway^54^. Genes of this novel pathway were not identified in *E.* sp. Strain 2 after sequence searches. Further experimental investigations may help elucidate whether the species might use another distinct strategy for l-ascorbate metabolism or might not uptake this nutrient at all.

### The pan-genome of *Erysipelothrix* genus shows a reduced accessory genome in the fish isolate 15TAL0474

**Figure 4:**
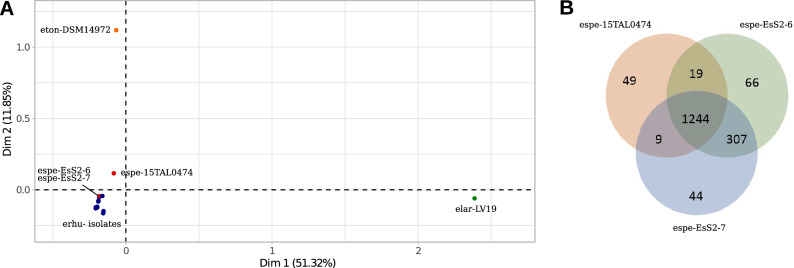
Pan-genome analysis of the genus *Erysipelothrix* and of *E.* sp. Strain 2 isolates. Legend: (**A**) Multiple correspondence analysis (MCA) of the genus *Erysipelothrix* pan-genome. Species mentioned in the figures are described in Supplementary Table S1. Blue dots belong to *E. rhusiopathiae* isolates. Red dots belong to *E.* sp. Strain 2 isolates. (**B**) Venn diagram of *E.* sp. Strain 2 pan-genome. Number of orthologous groups (OGs) that belong to the core genome (shared among the three isolates—union of all circles), number of accessory OGs (shared between two isolates—intersection of two circles), and number of singletons (exclusive/unique genes—remaining area of each circle) are described.

We examined the relationship among *Erysipelothrix* species based on a multiple correspondence analysis (MCA) of the pan-genome (Fig. 4A). *E. larvae* and *E. tonsillarum* were distantly related from the other most derived species, *E. rhusiopathia*e and *E.* sp. Strain 2, as expected (Fig. 4A). The most ancestral species are not only distantly related from the others based on the core protein sequence and whole nucleotide divergences (Fig. 2A,B, Fig. 3A,B), but also on gene content diversity (Fig. 4A). Surprisingly, *E.* sp. Strain 2 isolate 15TAL0474 was shown apart from the other two Strain 2 isolates (EsS2-6-Brazil and EsS2-7-Brazil), which fell within the *E. rhusiopathiae* group (Fig. 4A). Isolate 15TAL0474 shows the smallest proteome (1,352 protein coding genes) among all studied genomes (Supplementary Table S1). Thus, the core genome represents almost the totality (93.4%) of its proteome whereas for the other two related isolates (EsS2-6-Brazil and EsS2-7-Brazil), it comprises about 75% of their proteomes. This is likely a result of a reduced accessory genome (28 OGs) in isolate 15TAL0474 compared to the other two genomes (316 and 326 OGs) (Fig. 4B) and apparently, the missing set might explain the distance seen among these isolates in the MCA. Particularly, 15TAL0474 has 286 pseudogenes whereas EsS2-6-Brazil and EsS2-7-Brazil have only 21 and 16, respectively. In addition, among the 307 OGs shared between EsS2-6-Brazil and EsS2-7-Brazil, 293 OGs are also shared with *E. rhusiopathiae* group and most of them (~ 80% or 232/293 OGs) are consistently present in the accessory genome of *E. rhusiopathiae* (9 out of 10 strains), indicating that the accessory set was probably present in the core genome of the ancestral organism but has been under distinct pressures among strains. In addition to the missing accessory genes in 15TAL0474, the number of shared accessory genes between EsS2-strains and *E. rhusiopathiae* might explain their proximity in MCA.

We hypothesized that the extensive accessory reduction in 15TAL0474 could be related to an ongoing pseudogenization process. To check our hypothesis, we performed a reciprocal best hit (RBH) analysis of 15TAL0474 pseudogenes against the proteomes of all other genomes. A total of 200 (70%) pseudogenes had a RBH within the *E.* sp. Strain 2 group (with EsS2-6-Brazil and/or EsS2-7-Brazil) (Supplementary Table S7). Among them, 184 pseudogenes had hits with both EsS2-6-Brazil and EsS2-7-Brazil, and therefore, the core genome of *E.* sp. Strain 2 would be considerably raised from 1244 to 1428 OGs if the set of 15TAL0474 was functional. Genes related to transport and metabolism (carbohydrate [COG category G] and amino acid [E]) and information storage and processing (replication, recombination and repair [L]; translation, ribosomal structure and biogenesis [J]; transcription [K]) were the most represented (44.02% or 81/184) among decayed genes in 15TAL0474. The remaining pseudogenes had RBH with (1) only one EsS2-strain (16 pseudogenes), comprising the accessory genome of *E.* sp. Strain 2; (2) with itself (70 pseudogenes), comprising the exclusive set of 15TAL0474; or (3) with a gene outside the *E.* sp. Strain 2 group (16 pseudogenes). Therefore, the pan-genome analysis reveals the impact of gene reduction in 15TAL0474 as well suggesting the diverse genetic evolution for the pan- and core genomes among *Erysipelothrix* strains.

Genome downsizing has been shown in many bacterial species, which have undergone a transition from a free-living to a parasitic lifestyle. For instance, *Mycobacterium lepraemurium*^55^, *M. uberis*^56^, *Staphylococcus saccharolyticus*, *Shigella* spp.^57^, and *Rickettsia* spp.^58^ show reduced genomes that have been shrinking through gene decay and tend to minimize their gene content to the strictly required set as seen in *Mycoplasma genitalium*^59^. While the bacteria is adapting to the host niche, many genes become no longer major contributors for fitness in such environment and may be subject to gene decay. Since the host may provide required nutrients or machinery, genes of the core metabolism and DNA repair^57,58^ are commonly lost by the pathogen, which might explain their fastidious growth outside the host and mutation rate leading to pseudogenization. It is likely that *E.* sp. 15TAL0474 is under an ongoing reductive genome process to essentiality during its adaptation to a novel aquatic host whereas the orthologous genes remain needed in other isolates within the species, which colonize a distinct host. *E. rhusiopathiae* has also been described to have a wide-host spectrum^9^ as *E.* sp. Strain 2 and evidence of host-adapted strains are still scarce. Only recently, genetic determinants of *E. rhusiopathiae* strains were shown to be associated with pigs and wild boars, indicating host-associated strains^60^. We acknowledge that the small number of *E.* sp. Strain 2 isolates, including two epidemiologically related isolates, may not reflect the full genetic background of the species population and its diversity. Therefore, sequencing of further *E.* sp. Strain 2 isolates from distinct hosts might eventually help clarify the relationship between host and variants within this emerging species.

Here we reported a comprehensive comparative genomic analysis of the genus *Erysipelothrix.* Previous studies focused on *E. rhusiopathiae* whereas other species in the genus have been neglected. Thus, based on phylogenomics, and supported by dDDH and ANI values, we confirmed that the genus comprises a novel species, formerly known as *E.* sp. Strain 2, and recently named “*Erysipelothrix piscisicarius*”. We also showed that core functionalities shared by *E. rhusiopathiae* and *E.* sp. Strain 2 may be performed by homologous or analogous pathways, as illustrated by the C_4_-dicarboxylate transport. This reveals the complex biology of these organisms, which may employ distinct or alternative strategies to reach a similar purpose. Our work also uncovered distinct lineage-specific adaptations that have occurred within *E.* sp. Strain 2, resulting in a massive gene decay in the fish isolate. Considering the wide range of ecotypes in which *Erysipelothrix* species have been isolated, it is possible that a variety of survival strategies co‐evolved with the respective bacterial hosts. However, further studies are still needed to find out which selective forces might be acting over members of this novel clade isolated from distinct environments and also shaping their genomes. Finally, the findings reported here provide new insights into *Erysipelothrix* genome evolution and diversification that contribute to understanding the unique characteristics within the genus and may aid with new control strategies or prospective vaccine targets.

## Methods

### Whole genome sequencing

Two isolates of *Erysipelothrix* sp. Strain 2 from a farm turkey outbreak were randomly selected for whole genome sequencing and comparative genomics. Selected samples had been previously isolated from the lung and liver of deceased farm turkeys during the outbreak and confirmed as *Erysipelothrix* sp. Strain 2 by PCR, as described elsewhere^27^. Genomic DNA was extracted using Wizard Genomic DNA Purification kit (Promega,Wisconsin, USA) and quantified using Qubit HS dsDNA kit (Life Technologies, California, USA). DNA sequencing libraries were prepared using Illumina Nextera XT kit (Illumina, California, USA). Libraries were quantified and their quality was verified with Bioanalyzer (Agilent, California, USA). Whole genome sequencing was performed in a Illumina MiSeq platform (Illumina), using paired-end sequencing and 250 bp read length, which was conducted at the WEWSeq Biotecnologia (Curitiba, Brazil). Raw read quality was checked using FastQC^61^. Genomes were de novo assembled using SPAdes v. 3.12^62^ and annotated using NCBI Prokaryotic Genome Annotation Pipeline^63^.

### Comparative genomics

Comparative genome analyses were performed for a total of 15 *Erysipelothrix* genomes plus two outgroups belonging to the Erysipelotrichaceae family: *Holdemania filiformis* AF24-29 and *Turicibacter sp.* H121. In addition to our two *E.* sp. Strain 2 isolates (EsS2-6-Brazil and EsS2-7-Brazil), publicly available RefSeq genomes were retrieved from FTP-NCBI on December 14, 2018. At least one representative of *E. rhusiopathiae* clades (Clade 1, Clade 2 and Intermediate), according to Forde et al.^9^, were represented among selected genomes (Supplementary Material). Species and accession numbers for public available genomes used in this work are (Supplementary Table S1): *Erysipelothrix* sp. 15TAL0474 (NZ_CP034234.1), *E. rhusiopathiae* strains Fujisawa (NC_015601.1), NCTC8163 (NZ_LR134439.1), GXBY-1 (NZ_CP014861.1), ML101 (NZ_CP029804.1), WH13013 (NZ_CP017116.1), KC-Sb-R1 (NZ_CP033601.1), SY1027 (NC_021354.1), ATCC 19414 (NZ_ACLK00000000.2), NCTC7999 (NZ_UFYF00000000.1), and RU (NZ_RJTK00000000.1), *E. tonsillarum* DSM 14972 (NZ_AREO00000000.1), *E. larvae* LV19 (NZ_CP013213.1), *Holdemania filiformis* AF24-29 (NZ_QRUP01000001.1) and *Turicibacter* sp. H121 (NZ_CP013476.1). Genome accessions for *Erysipelothrix* sp. EsS2-6-Brazil and EsS2-7-Brazil, sequenced in this study, are: SBAR00000000.1 and SCFT00000000.1.

### Orthologous inference

FastOrtho software^64^ (https://github.com/olsonanl/FastOrtho) was used to define the orthologous groups. FastOrtho is a reimplementation of the OrthoMCL program^65^ that does not require the use of databases or Perl. Briefly, it uses BLASTP (v. 2.7.1+)^66^ to perform all-against-all homology search and also the MCL Markov Clustering algorithm^67^ to construct orthologous groups. BLASTP parameters were set as: -num_threads 7 -outfmt 7 -evalue 1e-05 -max_target_seqs 1000 and the remaining parameters were used as default. The MCL algorithm was used with default parameters.

### Functional annotation

Clusters of Orthologous Groups (COGs) were assigned to protein sequences using the Batch CD-Search online tool^68,69^ against the COG v1.0-4873 PSSMs database. COG annotations and functional categories (A-Z letter code) were attributed based on the most updated COG version^70^. Functional category enrichment analyses were calculated using the Fisher's exact test (*P* < 0.05). Pfam Domain annotations were obtained running hmmscan (v. 3.2.1) locally against the Pfam database release 32.0 (17,929 protein families)^71^ considering E-value ≤ 0.01. KEGG annotations were obtained from BlastKOALA^72^ and KofamKOALA^73^.

### Single-gene phylogenetic analysis

Single-gene phylogenetic trees were constructed using 16S rRNA gene and rpoB nucleotide sequences from 15 *Erysipelothrix* species with genomes available and from two outgroup species, *Holdemania filiformis* AF24-29 and *Turicibacter* sp. H121. For the 16S rRNA gene tree, sequences from *Erysipelothrix* sp. strain 715 and *E. inopinata* (whose genome sequences are not available to date) were included in the analysis. Sequences of these species were retrieved using an online BLASTN search^74^ with default parameters, using *E. rhusiopathiae* strain Fujisawa sequence as query. Sequences for each dataset were aligned with MUSCLE (v. 3.8.31)^75^ using default parameters, and poorly aligned columns were removed using trimAl (v. 1.4.rev22)^76^ with option -automated1. Best-fit nucleotide substitution models were selected using ModelTest-NG^77^ according to the corrected Akaike Information Criterion (AICc) implemented on Cipres Science Gateway^78^. Phylogenetic analyses were performed using Maximum Likelihood (ML) and Bayesian Analysis (BA) on Cipres Science Gateway^78^. ML search for the best-scoring ML tree was performed on RAxML (v. 8.2.12)^79^ under rapid bootstrap and stop bootstrap automatically (autoMRE) with majority rule criteria. BA analysis was performed on MrBayes (v. 3.2.7a)^80^, running two Markov Chain Monte Carlo (MCMC) runs of four chains each for 2,000,000 generations, sampling trees every 1000 generations with a burn-in of 25%. Phylogenetic trees were visualized and edited in FigTree (v. 1.4.2)^81^.

### Multilocus sequence analysis (MLSA)

MLSA phylogenetic tree was constructed based on the concatenated alignments of seven housekeeping genes (galK, gpsA, ldhA, prsA, pta, purA and recA) that have been previously proposed for multilocus sequence typing of *E. rhusiopathiae*^13^. Orthologous sequences for each individual genome were retrieved as previously described for 16S rRNA and rpoB. In addition, sequences from nine *Erysipelothrix* sp. Strain 2 isolated from fish (isolates 14TAL261U2, 14TAL260U1, 14TAL056U8, 14TAL259B, 15TAL055K2, 15TAL056U3, 15GAL055U1, 15TAL056K5, 14TAL259C) described elsewhere^26^ were included in this dataset. Sequences were aligned with MUSCLE (v. 3.8.31)^75^ and trimmed with trimAl (v. 1.4.rev22)^76^ as described above. Sequences were concatenated using FASconCAT-G (v. 1.04)^82^ and the best-fit partitioning schemes and nucleotide models of evolution were selected using PartitionFinder (v. 2.1.1)^83^ implemented on Cipres Science Gateway^78^. PartitionFinder settings used were: datatype = DNA, phylogeny program = raxml, branchlengths = linked, models = all, model_selection = aicc, search = all. Phylogenetic analyses were carried out using both ML and BA, under the respective partition schemes and models of evolution defined by PartitionFinder, with remaining parameters as described previously. Phylogenetic trees were visualized and edited in FigTree (v. 1.4.2)^81^.

### Phylogenomic analysis

Protein sequences of 618 single-copy core-genome orthologous groups from the 15 *Erysipelothrix* complete genomes were retrieved from the FastOrtho output file. We identified 112 genes potentially involved in horizontal gene transfer (HGT) events and removed their respective orthologous group (OG) to avoid their impact in the phylogenomic analysis (see details in the Supplementary Material). We ended up with a 506 OGs single-copy core genome dataset that was used to perform the phylogenomic analysis. For each individual orthologous group, sequences were aligned with MUSCLE (v. 3.8.31)^75^ and trimmed with trimAl (v. 1.4.rev22)^76^ as described above. The best-fit partitioning schemes and amino acid models of evolution were selected using PartitionFinder (v. 2.1.1)^83^ implemented on Cipres Science Gateway^78^, with the following settings: datatype = protein, phylogeny program = raxml, branchlengths = linked, models = all, model_selection = aicc, rcluster-max = 100, rcluster-percent = 10.0, search = rcluster^84^. Phylogenetic analyses were carried out using both ML and BA, under the respective partition schemes and models of evolution defined by PartitionFinder, with remaining parameters as described previously. Phylogenetic trees were visualized and edited in FigTree (v. 1.4.2)^81^.

### Analysis of pseudogenes in *Erysipelothrix* sp. 15TAL0474

In order to understand the evolution of pseudogenes in *Erysipelothrix* sp. 15TAL0474, putative amino acid sequences of the 286 pseudogenes (as annotated in the RefSeq version of the genome) were used as queries to run BLASTP (v. 2.7.1+)^65^ searches against the 15 *Erysipelothrix* complete genomes. For every query, the best hit in each distinct genome was retrieved to run a reciprocal BLASTP (v. 2.7.1+)^65^ against the genome of *Erysipelothrix* sp. 15TAL0474. When the best hit for the reciprocal BLASTP was the same initial pseudogene in Erysipelothrix sp. 15TAL0474, the two sequences were considered reciprocal best hits (RBH) and therefore, orthologous genes.

### Average nucleotide identity and digital DNA–DNA hybridization

The average nucleotide identity (ANI) and digital DNA–DNA hybridization (dDDH) values were calculated for all 17 genomes used in this study. ANI values were calculated for all pairwise comparisons using OrthoANIu algorithm^85^ available at the EzGenome web service^86^. Digital DDH values were calculated using the Genome-to-Genome Distance Calculator v. 2.1 available at the GGDC website service^41^. Matrices of ANI and dDDH values were visualized in heatmaps using Clustvis^87^, with a Manhattan distance calculation and a complete linkage for rows and columns.

### Ethical approval

This study was certified by the Animal Ethics Committee of Universidade Federal de Uberlândia, which was approved under the number A004/19. All procedures were performed in accordance with institutional guidelines and regulations of animal research.

## Supplementary Information

Supplementary Information 1.

Supplementary Information 2.

Supplementary Information 3.

Supplementary Information 4.

Supplementary Information 5.

Supplementary Information 6.

Supplementary Information 7.

Supplementary Information 8.

Supplementary Information 9.

Supplementary Information 10.

## Data Availability

The accession numbers for genomes used in this study are provided in Supplementary Table S1. Genome for de novo sequenced isolates *Erysipelothrix* sp. EsS2-6-Brazil and EsS2-7-Brazil will be made available upon publication of the manuscript.
