# Author Correction: Comparative genomics of a novel clade shed light on the evolution of the genus *Erysipelothrix* and characterise an emerging species

**DOI:** 10.1038/s41598-021-88892-3

**Published:** 2021-05-04

**Authors:** Ana Laura Grazziotin, Newton M. Vidal, Patricia Giovana Hoepers, Thais F. M. Reis, Dany Mesa, Luiz Felipe Caron, Max Ingberman, Breno C. B. Beirão, João Paulo Zuffo, Belchiolina Beatriz Fonseca

**Affiliations:** 1 Programa de Pós-Graduação em Ciências Veterinárias, Faculdade de Medicina Veterinária, Universidade Federal de Uberlândia, Rua Ceará, 1084, Bloco 2D, Sala 54, Umuarama, Uberlândia, MG CEP: 38405-240 Brasil; 2 Programa de Pós-Graduação em Biodiversidade Animal, Departamento de Evolução e Ecologia, Universidade Federal de Santa Maria, Avenida Roraima, 1000, Prédio 17, Sala 1140-D, Cidade Universitária, Bairro Camobi, Santa Maria, RS CEP: 97105-900 Brasil; 3 Departmento de Bioquímica e Biologia Molecular, Universidade Federal do Paraná, Curitiba, PR Brasil; 4 Departamento de Patologia Básica, Setor de Ciências Biológicas, Universidade Federal do Paraná, Curitiba, PR Brasil; 5 Imunova Análises Biológicas, Curitiba, PR Brasil; 6 Centro de Diagnóstico de Microbiologia Animal, Universidade do Estado de Santa Catarina, Florianópolis, SC Brasil

Correction to: *Scientific Reports* 10.1038/s41598-021-82959-x, published online 09 February 2021

The original version of this Article contained an error.

After publication it was brought to the authors' attention that according to the International Code of Nomenclature of Prokaryotes, the first taxonomically characterized and validated name for a species should be retained. Therefore, for E. sp. Strain 2 isolate 715, *Erysipelothrix piscisicarius* should be considered the official species name, rather than *E. takahashiae*.

In light of this, the text in the Abstract,

“Therefore, we confirm E. sp. Strain 2 represents a unique species that may be isolated from a broad host range, and the name “Erysipelothrix takahashiae” is suggested”

now reads:

“Therefore, we confirm E. sp. Strain 2 represents a unique species, that despite its official name “*Erysipelothrix piscisicarius”* (meaning a killer of fish), may be isolated from a broad host range”

In addition, the text in the Results and discussion,

“The International Code of Nomenclature of Prokaryotes^44^ recommends that when choosing a species name (Recommendation 12c) an epithet could indicate the source of the species or the name of the first discoverer. E. sp. Strain 2 has been isolated from a broad diversity of hosts, firstly from a pig (type strain 715)^23^, and then from fish (isolate 15TAL0474)^19^ and birds (isolates EsS2-6-Brazil and EsS2-7-Brazil)^27^. Given that the new species represents a pathogen of multiple distinct hosts (similarly to what is observed for *E. rhusiopathiae*) and that the name *E. piscisicarius* (meaning a killer of fish) does not represent the bacterium's full host spectrum, a more generic, unbiased name would be suitable. We suggest “*Erysipelothrix takahashiae”* after Toshio Takahashi who first discovered isolates of this clade and suggested it could represent a novel species^23^.”

now reads:

“The International Code of Nomenclature of Prokaryotes^44^ recommends that when choosing a species name (Recommendation 12c), isolates deemed conspecific should retain the species epithet provided on List of Prokaryotic names with Standing in Nomenclature. E. sp. Strain 2 has been isolated from a broad diversity of hosts, firstly from a pig (type strain 715)^23^, and then from fish (isolate 15TAL0474)^19^ and birds (isolates EsS2-6-Brazil and EsS2-7-Brazil)^27^. Nevertheless, though the new species represents a pathogen of multiple distinct hosts (similarly to what is observed for *E. rhusiopathiae*), and the name *E. piscisicarius* (meaning a killer of fish) does not represent the bacterium's full host spectrum, as the first taxonomically characterized and validated name for the species^19^, “*Erysipelothrix piscisicarius”* should be considered the official species name for E. sp. Strain 2.”

And the text,

Thus, based on phylogenomics, and supported by dDDH and ANI values, we confirmed that the genus comprises a novel species, E. sp. Strain 2, for which we suggest the name “Erysipelothrix takahashiae”.”

now reads:

“Thus, based on phylogenomics, and supported by dDDH and ANI values, we confirmed that the genus comprises a novel species, formerly known as E. sp. Strain 2, and recently named “*Erysipelothrix piscisicarius*”.”

These changes have been implemented in the PDF and HTML versions of the Article, and do not affect the findings and conclusion.

