# Supplementary material for: Comparative genomics of a novel clade shed light on the evolution of the genus Erysipelothrix and characterise an emerging species
Source: Sci Rep. 2021 Feb 9;11:3383. doi: 10.1038/s41598-021-82959-x (PMC7873064; doi:10.1038/s41598-021-82959-x)
Supplement: Supplementary file 10 — Supplementary Information 10. [file 41598_2021_82959_MOESM10_ESM.pdf]

SUPPLEMENTARY INFORMATION FOR

Comparative genomics of a novel clade shed light on the evolution of the genus *Erysipelothrix* and characterise an emerging species

Ana Laura Grazziotin, Newton M. Vidal, Patricia Giovana Hoepers, Thais F. M. Reis, Dany Mesa, Luiz Felipe Caron, Max Ingberman, Breno C. B. Beirão, João Paulo Zuffo, Belchiolina Beatriz Fonseca

Contents:

- Supplementary material
- Supplementary results and discussion
- Supplementary figures
- Caption and legend of Supplementary tables provided as individual spreadsheets
- References cited in this supplementary file

SUPPLEMENTARY MATERIAL

Identification of Spa protein sequences in *Erysipelothrix* isolates used in this study: To identify Spa protein sequences in the *Erysipelothrix* genomes, we retrieved reference *spa* gene sequences for each molecular type, *spaA* (accession: AB259652), *spaB* (AB238211) and *spaC* (AB238210), as previously described (To & Nagai, 2007) from NCBI. Spa translated sequences were used as query sequences in BLASTP and TBLASTN searches against *Erysipelothrix* proteomes and genomes used in this study, respectively. A representative protein sequence of each molecular type was chosen, in addition to the novel type found in *E. tonsillarum*, and were aligned using MUSCLE (v. 3.8.31) (Edgar, 2004) for sequence comparison. Pairwise identities and similarities were calculated using the program Needle available in the EMBOSS package (Rice et al., 2000).

Identification of *E. rhusiopathiae* clades among RefSeq genomes used in this study: The intraspecific variability among *E. rhusiopathiae* strains was previously identified and four distinct *E. rhusiopathiae* clades were proposed by Forde et al., 2016. In this work, we used 10 RefSeq genomes publicly available for *E. rhusiopathiae* and checked to which clade they belong to. Based on Forde et al., 2016, clades were already known for four genomes used in this study: ATCC19414 (Clade 2) and Fujisawa, SY1027 and GXBY-1 (Clade Intermediate), but for the other 6 genomes clades were

unknown. Therefore, we selected one or two representative isolates from each clade, as previously identified (Forde et al., 2016) as follow: Clade 1 (isolates P-92 and Baño 36); Clade 2 (isolates 2197-i and 266); Clade Intermediate (isolates Grizzly) and Clade 3 (Kaparek and Mew-22). Reads of each representative genome from Forde et al., 2016 were retrieved from SRA (Sequence Reads Archive) and assembled using SPAdes v.3.12 (Bankevich et al., 2012). The relatedness among all 17 *E. rhusiopathiae* genomes was checked using two whole genome nucleotide metrics, dDDH (digital DNA:DNA hybridization)(Meier-Kolthoff et al., 2013) and ANI (Average Nucleotide Identity)(Lee et al., 2016; Yoon et al., 2017), and resulting identities were plotted in a heatmap using Clustvis (Metsalu & Vilo, 2015). *E. rhusiopathiae* genomes used in this study belong to the following clades: Clade 1 (KC-Sb-R1), Clade 2 (RU, ATCC19414 and NCTC8163) and Intermediate (Fujisawa, SY1027, GXBY-1, ML101 and NCTC7999)(Supplementary Figure S2).

Identification of single-copy core genes potentially involved in horizontal gene transfer: We identified genes potentially involved in horizontal gene transfer (HGT) events and removed their respective orthologous group (OG) from the single-copy core genome dataset to prevent bias in the phylogenomic analysis. Protein sequences from *E. rhusiopathiae* Fujisawa (one of the most derived species) and *E. larvae* (the most ancient species) were used as query sequences to run BLASTP searches (parameters: -max_target_seqs 100 -remote) against the NR database at the NCBI. Results were tabulated and manually inspected. When a query sequence had a better hit (considering both E-value and identity) to a sequence belonging to other genera rather than *Erysipelothrix*, this sequence was considered potentially involved in HGT events. All queries (618 sequences) from *E. rhusiopathiae* retrieved best hits belonging to *Erysipelothrix* species before retrieving hits against any other genera, showing no putative genes involved in HGT events. A hundred and twelve queries out of 618 from *E. larvae* had a better hit against other genera rather than *Erysipelothrix.* After removing the respective 112 OGs from the single-copy core genome dataset, the phylogenomic analysis was constructed based on 506 OGs single-copy core genome dataset.

Identification of the accessory genes shared only by *E.* sp. EsS2-6-Brazil and *E.* sp. EsS2-7-Brazil in isolate *E.* sp. 15TAL0474: A total of 307 sequences belong to the accessory genome of the turkey isolates (i.e., shared between *E.* sp. EsS2-6-Brazil and EsS2-7-Brazil) but were found missing in the fish isolate genome (*E.* sp. 15TAL0474). To verify that the genes were not missed due to misannotation in *E.* sp. 15TAL0474, we performed BLASTP and TBLASTN searches of either *E.* sp. EsS2-6-Brazil and EsS2-7-Brazil protein sequences (as queries) against the *E.* sp. 15TAL0474 pseudogenes and

genome, respectively. BLAST results were classified according to Blanc et al. (2007) as follow: *unannotated coding gene* – when a hit found using TBLASTN was >50% of the query coverage; *annotated pseudogene* – when a hit was found using BLASTP against an annotated pseudogene; *unannotated pseudogene* – when a hit found using TBLASTN had a E-value better than 0.01 and HSP > 20 amino acids or query coverage >20% ; *absent* – when no hit was found with previous criteria. No *unannotated coding genes* were found in *E.* sp. 15TAL0474. After searches, we found that 197 queries out of 307 OGs were found in 15TAL0474 as annotated pseudogenes, 47/307 OGs were found as unannotated pseudogenes, and 63/307 OGs had no hits in 15TAL0474 and were considered absent (Supplementary Table S9). Therefore, the missed gene set of *E.* sp. 15TAL0474 is not related to faulty annotation.

SUPPLEMENTARY RESULTS AND DISCUSSION

General genome characteristics

Sequenced isolates (*E.* sp. EsS2-6-Brazil and *E.* sp. EsS2-7-Brazil) had been previously confirmed as *E.* sp. Strain 2-related isolates (Hoepers et al., 2019) based on a PCR method developed elsewhere (Pal et al., 2010). The WGS generated 3,739,346 total sequenced reads for isolate EsS2-6-Brazil and 2,914,734 for EsS2-7-Brazil, corresponding to approximately 431x and 322x genome coverage, respectively. The draft genome sequences were assembled in 30 contigs each, comprising a total of 1,739,138 bp and 1,714,240 bp for isolate EsS2-6-Brazil and EsS2-7-Brazil, respectively, making them amongst the smallest genome sizes compared to other *Erysipelothrix* spp. genomes available (Supplementary Table S1).

Estimating the genus core genome

To help us understand the phylogenetic relationship and bacterial evolution of the genus, we used the program FastOrtho to predict orthologous groups (OGs) among *Erysipelothrix* species. Considering the four species (*E. larvae*, *E. tonsillarum*, *E. rhusiopathiae* and *E.* sp. Strain 2) of the genus, FastOrtho predicted a total of 2,007 OGs and 1,138 orphans revealing a pan-genome of 3,145 protein families. The genus core genome consisted of 647 OGs, of which 618 were represented by single-copy genes. A total of 112 OGs were disregarded due to possible horizontal gene transfer (HGT) events in *E. larvae* (Supplementary Material). The core genome represents 18.5% and 30.9% of the protein coding gene content for *E. larvae* and *E. tonsillarum*, respectively, and 34% of the average gene content across *E. rhusiopathiae* and *E.* sp. Strain 2. Among the protein families found in all *E. rhusiopathiae* and *E.* sp. Strain 2 isolates, but *E. larvae* and *E. tonsillarum*, are several copies (average 5.7 copies) of IS*30*

family transposase (Kegg entry K07482), alanine-glyoxylate aminotransferase (no Kegg entry) and CDP-glycerol-glyverophosphate glycerophosphotransferase (K09809). In contrast, protein families such as group II intron reverse transcriptase/maturase (K00986), type I restriction endonuclease subunit R (K01153), MgtC/SapB family protein (K07507), 6-phospho-alpha-glucosidase (K01232) were only found in *E. larvae* and *E. tonsillarum* (Supplementary Table S8).

Unique repertoire of *Erysipelothrix* species reveals exclusive expansions in *E. larvae*

Unique genes (strain-specific genes) were found in most studied genomes. *E.* sp. Strain 2 isolates showed 35 (isolate 15TAL0474), 41 (isolate EsS2-6-Brazil) and 29 (isolate EsS2-7-Brazil) exclusive genes. Among *E. rhusiopathiae* genomes, few genes comprise the exclusive set, indicating that the vast majority of the gene content is shared at some level among distinct clades (Forde et al., 2016) within the species (Supplementary Fig. 2, Supplementary Fig. 6 and Supplementary Methods). The considerable number of shared genes (core and accessory) in addition to the low abundance of unique sets in *E. rhusiopathia*e (Supplementary Fig. 6) is likely to be caused by genetic homogeneity among strains rather than a lack of representativeness, once isolates from three distinct clades were considered (Supplementary Fig. 2).

As expected, *E. larvae* and *E. tonsillarum* showed the highest number of unique genes (1,090 and 185 genes, respectively), which is likely a consequence of i) the lack of other available genomes from the same species, which would demonstrate the repertoire of shared genes (probably including many genes of the current unique list (Supplementary Table S8)) within the species and ii) being the two most ancient species (Fig. 2B) and distantly related (Fig. 3AB) to *E. rhusiopathiae* and *E.* sp. Strain 2 genomes. Surprisingly, *E. larvae* showed a considerable fraction (27.3% or 298/1090 genes) of lineage-specific expansions in its exclusive gene set. In constrast, *E. tonsillarum* showed only few instances (about 2.2% or 4/185 genes) of lineage-specific expansions in its exclusive gene set. Most of the expanded unique families in *E. larvae* were poorly characterized sequences related to the mobilome, such as chromosomal transposases and insertion sequences. Interestingly, protein family 6-phospho-beta-glucosidase (K01223; EC: 3.2.1.86) represents a lineage-specific expansion of *E. larvae*. This protein family is involved in starch and sucrose metabolism (Ko00500) responsible for the hydrolysis of cellobiose-6-phosphate (imported as cellobiose from extracellular environment) to D-glucose. *E. larvae* was isolated from the healthy gut microbiota of a rhinoceros beetle, *Trypoxylus dichotomus* (Bang et al., 2014), which is fed on decayed wood. Therefore, the expansion of a protein related to cellulose degradation represents an important advantage not only to the bacteria but also to the host insect, since it promotes efficient digestion of the disaccharide.

SUPPLEMENTARY FIGURES

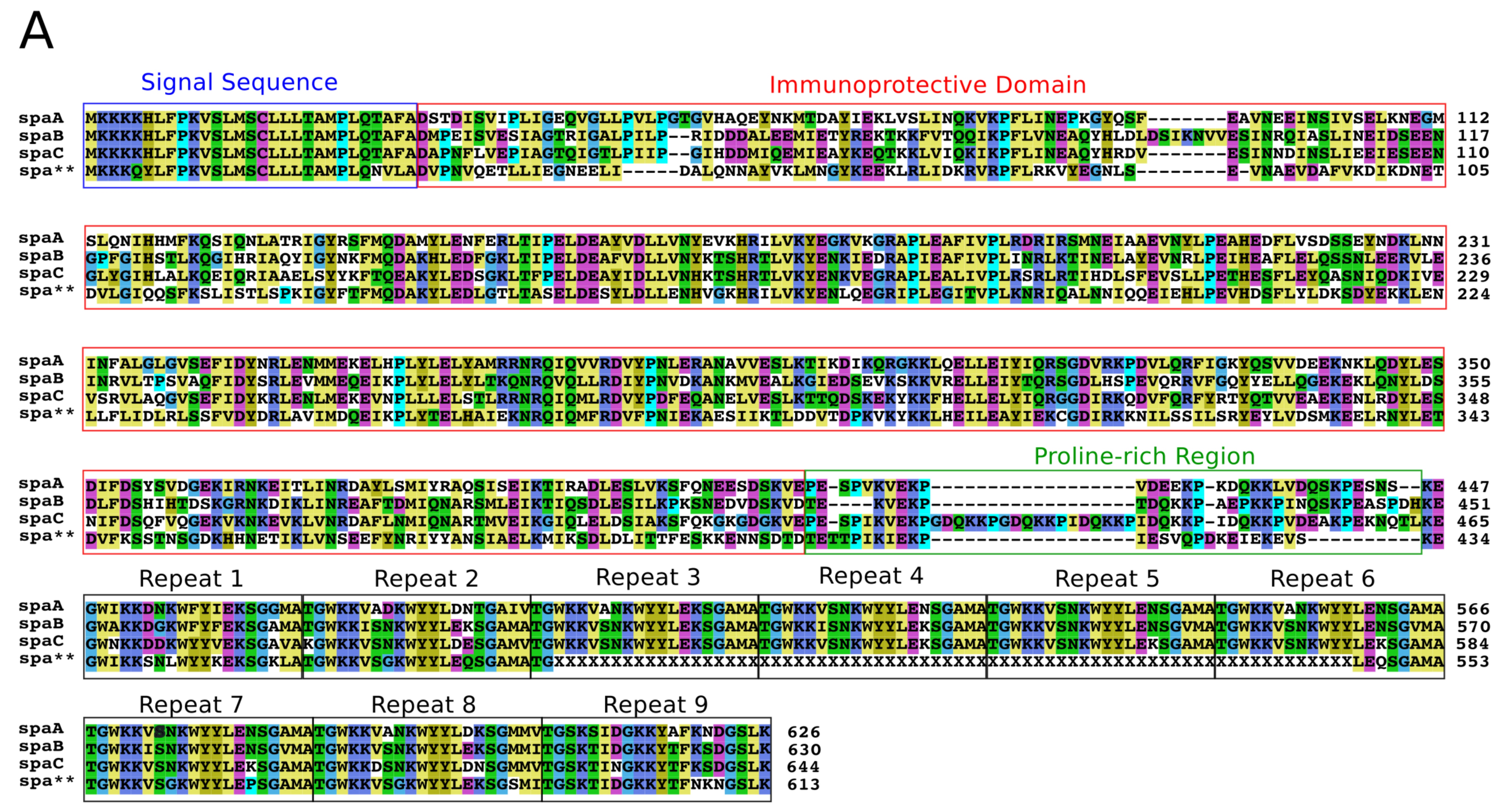

B

spa protein	spaA	spaB	spaC	spa**	
**spaA**	100.0%	62.2%	63.9%	**43.8%**	Identity
**spaB**	78.4%	100.0%	65.9%	**41.1%**	
**spaC**	78.8%	79.7%	100.0%	**37.9%**	
**spa****	**62.3%**	**58.2%**	**53.4%**	100.0%	
	Similarity				

Supplementary Figure S1 – Sequence analysis of Spa proteins in *Erysipelothrix*.
Legend: (A) Multiple sequence alignment of the four distinct types of Spa proteins found in *Erysipelothrix* genera. Colored boxes indicate the domain structure of the protein: red - signal sequence, blue - immunoprotective domain, green - proline-rich region, black - ~20-amino-acid repeat domain. Background colours in the alignment represent similar amino acids. Sequence names: SpaA - *E. rhusiopathiae* Fujisawa, SpaB - *E. rhusiopathiae* KC-Sb-R1, SpaC - *E.* sp. Strain 2 isolate EsS2-6-Brazil, Spa** - new Spa protein found in *E. tonsillarum* DSM 14972. (B) Matrix of identity (upper diagonal) and similarity (lower diagonal) among the distinct types of Spa proteins. Values compared to the new Spa protein found in *E. tonsillarum* are in bold.

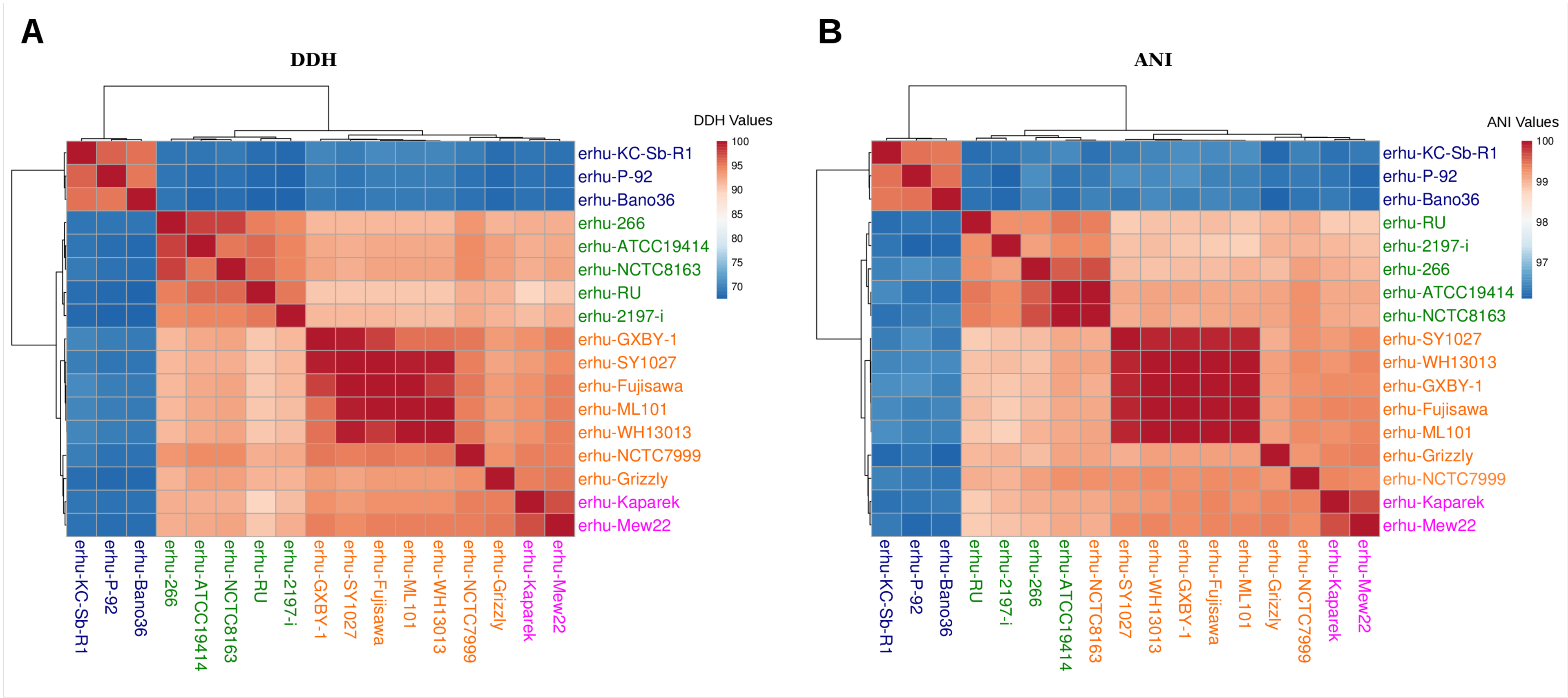

Supplementary Figure S2 – Heatmap of whole-genome sequence pairwise comparisons between *E. rhusiopathiae* isolates from RefSeq and from assigned Clades according to Forde et al., 2016.

Legend: (A) Heatmap of the digital DNA:DNA hybridizations (dDDH). (B) Heatmap of the Average Nucleotide Identity (ANI). Species abbreviations are color-coded according their Clades: Clade 1 – blue; Clade 2 – green; Clade Intermediate – Orange and Clade 3 – pink. Abbreviations for RefSeq genomes used in this study are also described Supplementary Table S1.

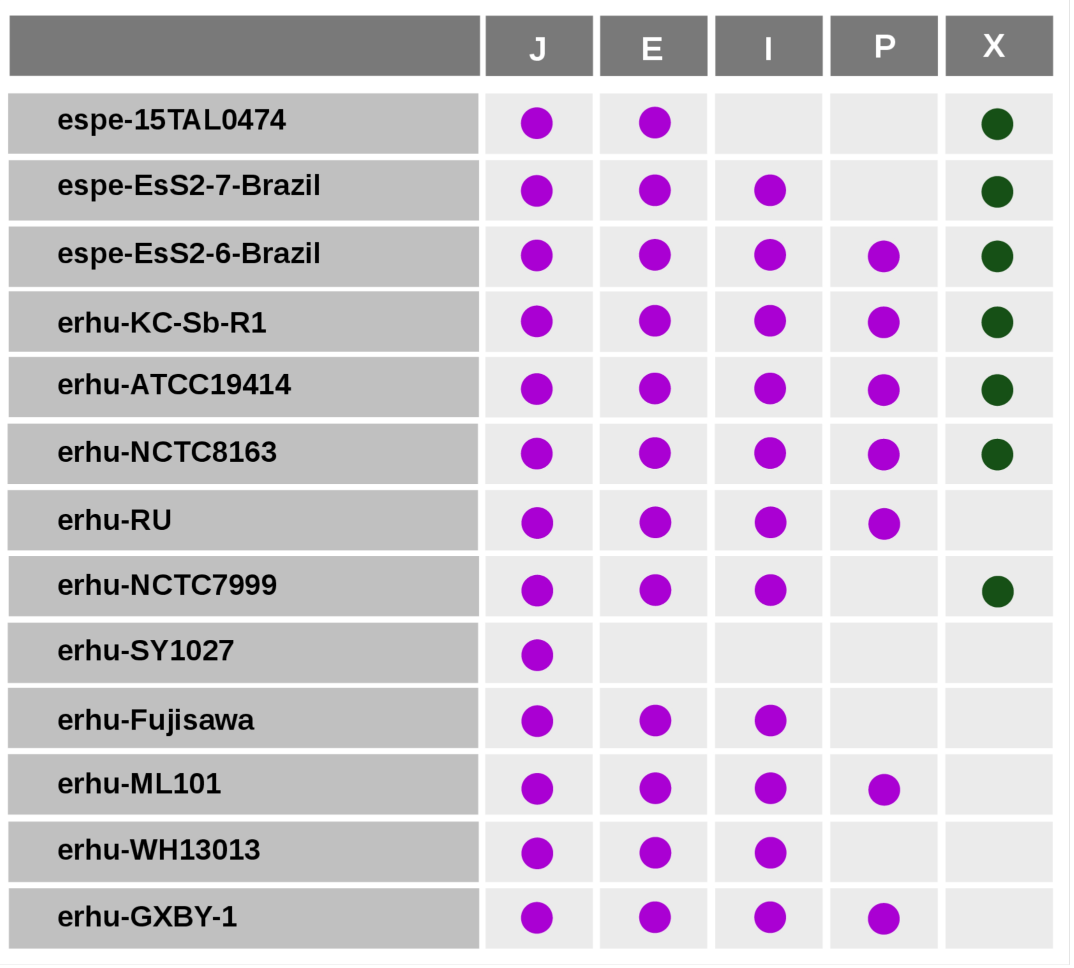

Supplementary Figure S3 – Functional enrichment analysis of the core genome shared between *E. rhusiopathiae* and *E.* sp. Strain 2 isolates.
Legend: Circles in magenta represent core genome-enriched categories and circles in green represent accessory genome-enriched categories (Fisher's Exact Test; $P < 0.05$). Enriched COG categories are: J – Translation, ribosomal structure and biogenesis, E – Amino acid transport and metabolism, I – Lipid transport and metabolism, P – Inorganic ion transport and metabolism, and X - Mobilome: prophages, transposons. Genes of core genome-enriched categories are described in Supplementary Table S4.

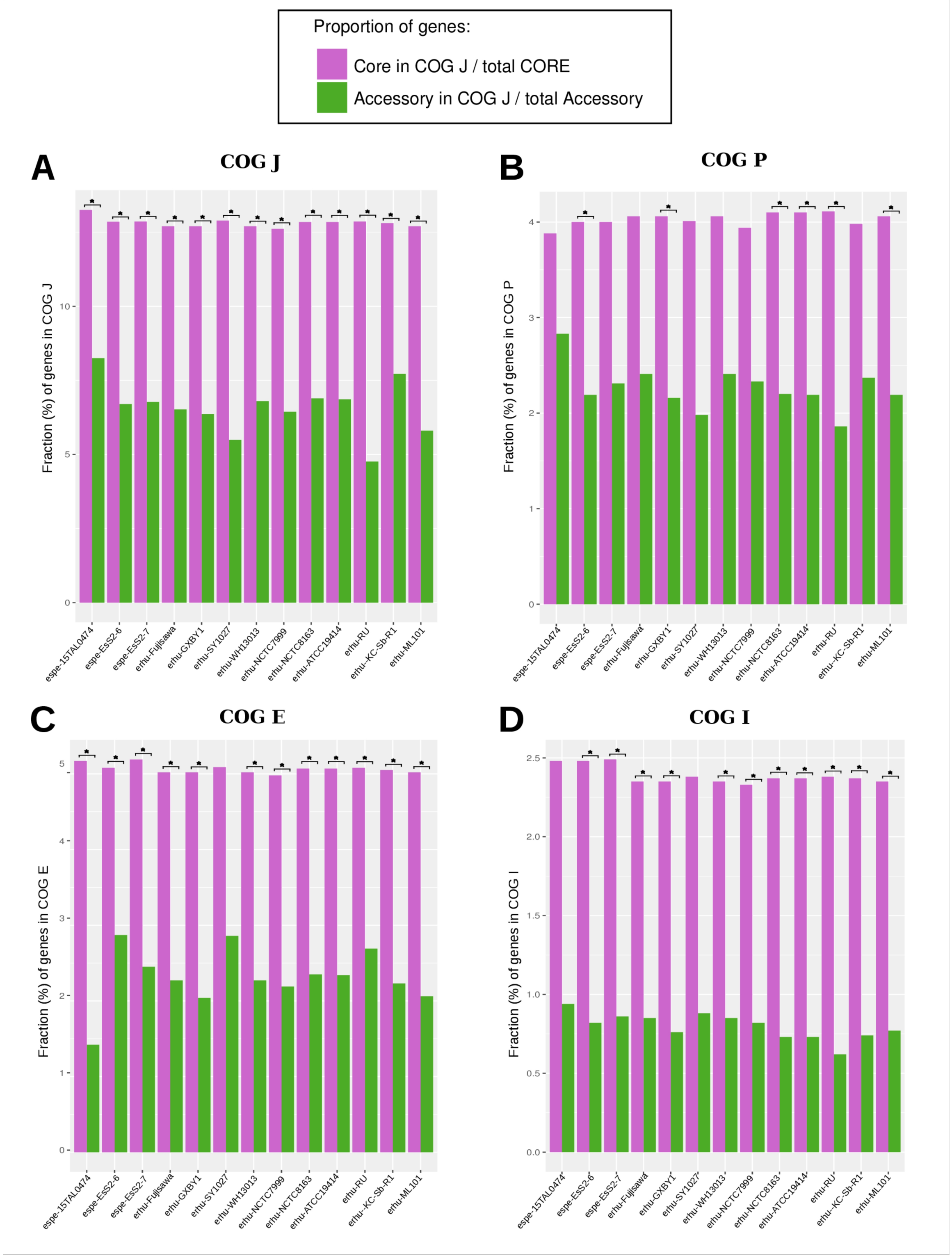

Supplementary Fig. S4 – Cluster of Orthologous Groups (COGs) categories overrepresented in the core genome of *E. rhusiopathiae* and *E.* sp. Strain 2 isolates.

Legend: (A) COG J - Translation, ribosomal structure and biogenesis, (B) COG P – Inorganic ion transport and metabolism, (C) COG E – Amino acid transport and metabolism, and (D) COG I – Lipid transport and metabolism. A COG category was considered enriched when p-value < 0.05 (Fisher's exact test) and is indicated by asterisks (Supplementary Table S5). Organism abbreviation is described in Supplementary Table S1.

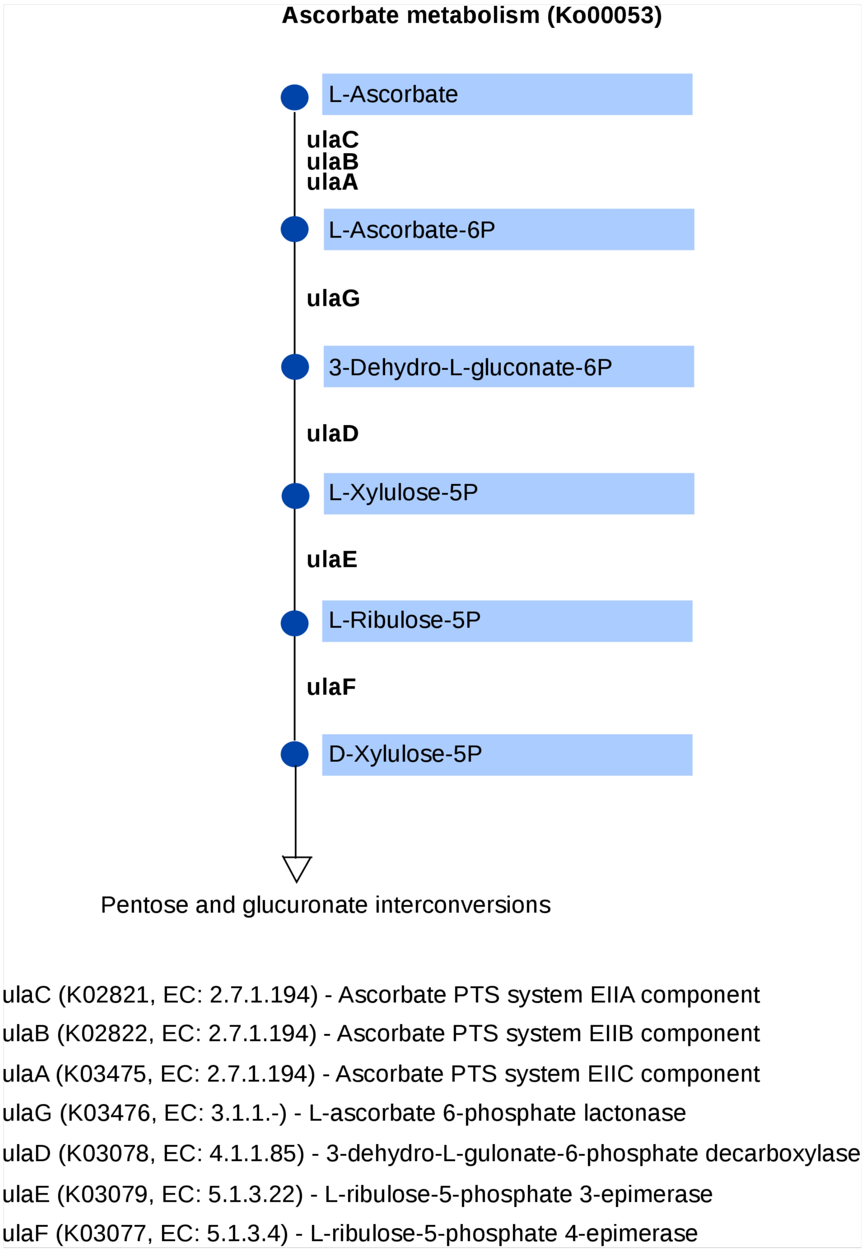

Supplementary Fig. S5 – Ascorbate metabolic pathway present in core genome of *E. rhusiopathiae*.
Legend: Genes (locus tag) related to each enzyme are: **ulaC** (HMPREF0357_RS01330, ERH_RS03675, A2I91_RS05890, EEY85_RS05380, DM789_RS03700, DYB49_RS03295, EL194_RS06865, EF876_RS02400, K210_RS01290, BC346_RS05410), **ulaB** (HMPREF0357_RS01340, ERH_RS03685, A2I91_RS05880, EEY85_RS05370, DM789_RS03710, DYB49_RS03305, EL194_RS06875, EF876_RS02390, K210_RS01300, BC346_RS05400), **ulaA** (HMPREF0357_RS01335, ERH_RS03680, A2I91_RS05885, EEY85_RS05375, DM789_RS03705, DYB49_RS03300, EL194_RS06870, EF876_RS02395, K210_RS01295, BC346_RS05405), **ulaG** (HMPREF0357_RS01325, ERH_RS03670, A2I91_RS05895, EEY85_RS05385, DM789_RS03695, DYB49_RS03290, EL194_RS06860, EF876_RS02405, K210_RS01285, BC346_RS05415), **ulaD** (ERH_RS03690, A2I91_RS05875, DM789_RS03715, DYB49_RS03310, EL194_RS06880, BC346_RS05395, HMPREF0357_RS01345, EEY85_RS05365), **ulaE** (ERH_RS03695, A2I91_RS05870, DM789_RS03720, DYB49_RS03315, EL194_RS06885, BC346_RS05390, HMPREF0357_RS01350, EF876_RS02380, EEY85_RS05360), **ulaF** (HMPREF0357_RS01355, ERH_RS03700, A2I91_RS05865, EEY85_RS05355, DM789_RS03725, DYB49_RS03320, EL194_RS06890, EF876_RS02375, K210_RS01315, BC346_RS05385) and are described in Supplementary Table S6, except ulaE and ulaF because they are pseudogenes in strains SY1027 and RU. Figure was based on KEGG Database (Kanehisa & Goto, 2000).

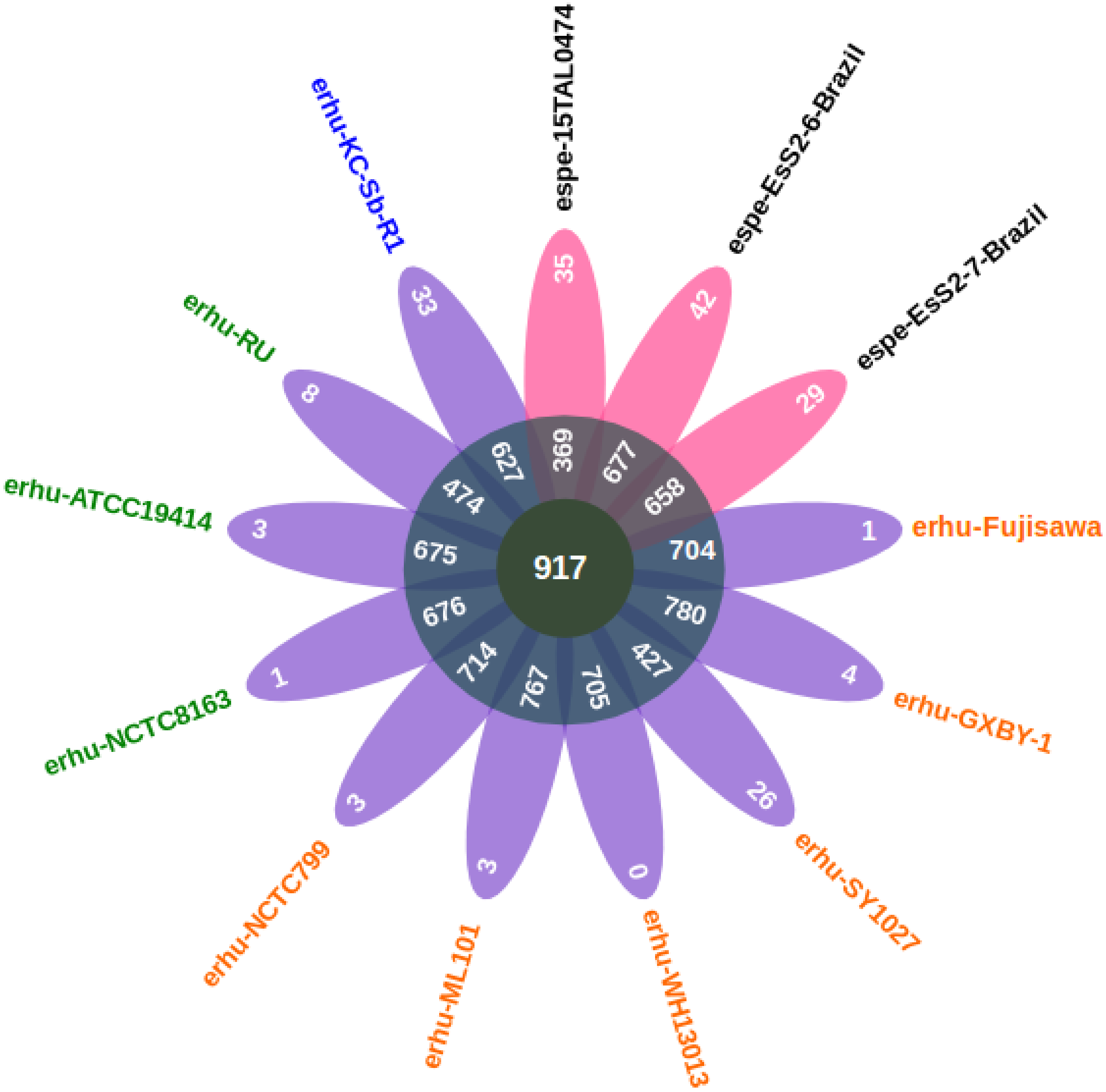

Supplementary Fig. S6 – A flower plot of the pan-genome of *E. rhusiopathiae* and *E.* sp. Strain 2 isolates. Legend: A flower-plot schematic representation illustrates the number of predicted core (917) and accessory (369 to 780) genes among *E. rhusiopathiae* and *E.* sp. Strain 2 isolates. Petals show the number of predicted unique genes for each isolate. *E. rhusiopathiae* isolates are color-coded according their Clades (Forde et al., 2016): Clade 1 – blue; Clade 2 – green; Clade Intermediate – Orange. *E. rhusiopathiae* and *E.* sp. Strain 2 isolates were indicated as described in Supplementary Table S1.

SUPPLEMENTARY TABLES

Table captions and legends are provided in this document whereas data are available in spreadsheet tables.

Supplementary Table S1 – General information regarding genomes selected for this study.

Supplementary Table S2 – Matrix of identities (%) for rpoB and 16S rRNA gene sequences (BLASTn).

Legend: *E.* sp. Strain 2 isolates were highlighted in red whereas *E. rhusiopathiae* isolates were highlighted in cyan. The standard threshold value used as species boundaries for 16S rRNA and rpoB sequences is 97% (Stackebrandt & Goebel, 1994) and 97.7% (Adékambi et al., 2003; Adékambi et al., 2006), respectively. Organism abbreviation is described in Supplementary Table S1. In addition, organism abbreviations not shown in Supplementary Table S1 (isolates with no genome available until submission of this study) are: *Erysipelothrix inopinata* 143-02 (eino-143-02) and *Erysipelothrix* sp. 715 (espe-715).

Supplementary Table S3 – Matrix of digital DNA:DNA hybridization (dDDH) and Average Nucleotide Identity (ANI) values (%).

Legend: *E.* sp. Strain 2 isolates were highlighted in red whereas *E. rhusiopathiae* isolates were highlighted in cyan. The established same-species delineation thresholds are 70% for dDDH (Auch et al., 2010; Meier-Kolthoff et al., 2013) and 95% for ANI (Goris et al., 2007) values. Organism abbreviation is described in Supplementary Table S1.

Supplementary Table S4 – Enrichment analysis of COG categories in *E. rhusiopathiae* and *E.* sp. Strain 2 genomes.

Legend: Functional category enrichment analysis was calculated using the Fisher's exact test. The p-value, followed by the odds value (between parentheses), is shown in each cell. A p-value < 0.05 was considered significant. An odds value greater than 1.0 indicates a COG category enriched in the core genome (highlighted in pink), whereas an odds value lower than 1.0 indicates a COG category enriched in the accessory genome (highlighted in green).

Supplementary Table S5 – List of genes that belong to the enriched Cluster of Orthologous Groups (COG) categories E, I and P in the core genome of *E. rhusiopathiae* and of *E.* sp. Strain 2 isolates.

Legend: COG E – Amino acid transport and metabolism, COG I – Lipid transport and metabolism, and COG P – Inorganic ion transport and metabolism. Rows were colored according to the COG category a gene belongs to: red for COG E, blue for COG I, and yellow for COG P. Column "Core/accessory/unique" shows whether a gene belongs to the core genome (shared among all genomes) or accessory genome (shared between two or more, but not all genomes) or unique (exclusive of one genome) inferred amongst *E. rhusiopathiae* isolates (core-erhu, accessory-erhu, unique-erhu) and amongst *E.* sp. Strain 2 isolates (core-strain2, accessory-strain2, unique-strain2). A cell content showing "NA" (Not Assigned) means that no KO number from Kyoto Encyclopedia of Genes and Genomes (KEGG), KO number description (KEGG), Pfam domain, Pfam domain architecture, E-value (Pfam) was found in the analysis. Organism abbreviation is described in Supplementary Table S1.

Supplementary Table S6 – List of core genes identified in *E. rhusiopathia*e isolates and core genes identified in *E.* sp. Strain 2 isolates.

Legend: Column "CORE erhu or strain2" shows whether a gene belongs to the core genome of *E. rhusiopathiae* isolates (core-erhu) or *E.* sp. Strain 2 isolates (core-strain2). A cell content showing "NA" (Not Assigned) means that no Cluster of Orthologous Groups (COG) or COG category was found in the analysis. Organism abbreviation is described in Supplementary Table S1.

Supplementary Table S7 – List of pseudogenes in *Erysipelothrix* sp. 15TAL0474 with reciprocal best hit against the 15 *Erysipelothrix* complete genomes.

Legend: Organism abbreviation is described in Supplementary Table S1. HSP: High-scoring Segment Pair.

Supplementary Table S8 – List of unique genes in *E. tonsillarum* and *E. larvae.*

Legend: A cell content showing "NA" (Not Assigned) means that no Cluster of Orthologous Groups (COG) or COG category was found in the analysis. Organism abbreviation is described in Supplementary Table S1.

Supplementary Table S9 – Sequence search of *Erysipelothrix* sp. EsS2-6-Brazil and EsS2-7-Brazil accessory genes against *Erysipelothrix* sp. 15TAL0474.

Legend: BLAST hits were considered pseudogenes when E-value was better than 0.01 and query coverage > 20% of the protein length according to (Blanc et al., 2007). Column “Status in *Erysipelothrix* sp. 15TAL0474” shows whether a BLAST search of an accessory gene (Query Locus-tag) from *E.* sp. EsS2-6-Brazil or EsS2-7-Brazil recovered a previously “annotated pseudogene” or a previously “unannotated pseudogene” in *E.* sp. 15TAL0474 or had no hits (“absent”) in *E.* sp. 15TAL0474. Organism abbreviation is described in Supplementary Table S1.

REFERENCES

Adékambi, T., Berger, P., Raoult, D. & Drancourt, M. rpoB gene sequence-based characterization of emerging non-tuberculous mycobacteria with descriptions of *Mycobacterium bolletii* sp. nov., *Mycobacterium phocaicum* sp. nov. and *Mycobacterium aubagnense* sp. nov. *Int J Syst Evol Microbiol* **56**, 133–143 (2006).

Adékambi, T., Colson, P. & Drancourt, M. rpoB-based identification of nonpigmented and late-pigmenting rapidly growing mycobacteria. *J Clin Microbiol* **41**, 5699–5708 (2003).

Auch, A. F., von Jan, M., Klenk, H. P. & Göker, M. Digital DNA-DNA hybridization for microbial species delineation by means of genome-to-genome sequence comparison. *Stand Genomic Sci* **2**, 117-34 (2010).

Bang, B. H., Rhee, M. S., Chang, D. H., Park, D. S. & Kim, B. C. *Erysipelothrix larvae* sp. nov., isolated from the larval gut of the rhinoceros beetle, *Trypoxylus dichotomus* (Coleoptera: Scarabaeidae). *Antonie Van Leeuwenhoek* **107**, 443-451 (2014).

Bankevich, A. et al. SPAdes: A New Genome Assembly Algorithm and Its Applications to Single-Cell Sequencing. *J Comput Biol* **19**:455-77 (2012).

Blanc, G. et al. Reductive genome evolution from the mother of *Rickettsia*. *PLoS Genet* **3**, e14 (2007) .

Edgar, R. C. MUSCLE: multiple sequence alignment with high accuracy and high throughput. *Nucleic Acids Res* **32**, 1792-1797 (2004).

Forde, T. et al. Genomic analysis of the multi-host pathogen *Erysipelothrix rhusiopathiae* reveals extensive recombination as well as the existence of three generalist clades with wide geographic distribution. *BMC Genomics* **17**, 461; 10.1186/s12864-016-2643-0 (2016).

Goris, J. et al. DNA-DNA hybridization values and their relationship to whole-genome sequence similarities. *Int J Syst Evol Microbiol* **57**, 81-91 (2007).

Hoepers, P. G. et al. First outbreak reported caused by *Erysipelothrix* species strain 2 in turkeys from poultry-producing farms in Brazil. *Annals of Microbiology* **69**, 1211–1215 (2019).

Kanehisa, M. and GoTo, S. KEGG: Kyoto Encyclopedia of Genes and Genomes. *Nucleic Acids Res* **28**, 27-30 (2000).

Lee, I., Ouk Kim, Y., Park, S. C. & Chun, J. OrthoANI: An improved algorithm and software for calculating average nucleotide identity. *Int J Syst Evol Microbiol* **66**, 1100-1103 (2016).

Meier-Kolthoff, J. P., Auch, A. F., Klenk, H. & Göker, M. Genome sequence-based species delimitation

with confidence intervals and improved distance functions. *BMC Bioinformatics* **21**, 14-60 (2013).

Metsalu, T. & Vilo, J. ClustVis: a web tool for visualizing clustering of multivariate data using Principal Component Analysis and heatmap. *Nucleic Acids Res* **43**, W566-W570 (2015).

Pal, N., Bender, J. S., Opriessnig, T. Rapid detection and differentiation of *Erysipelothrix* spp. by a novel multiplex real-time PCR assay. *J Appl Microbiol* **108**, 1083-1093 (2010).

Rice, P., Longden, I. & Bleasby, A. EMBOSS: The European Molecular Biology Open Software Suite. *Trends Genet* **16**, 276-277 (2000).

Stackebrandt, E. & Goebel, B. Taxonomic note: a place for DNA-DNA reassociation and 16S rRNA sequence analysis in the present species definition in bacteriology. *Int J Syst Evol Microbiol* **44**, 846-849 (1994).

To, H. & Nagai, S. Genetic and antigenic diversity of the surface protective antigen proteins of *Erysipelothrix rhusiopathiae*. *Clin Vaccine Immunol* **14**, 813-820 (2007).

Yoon, S.H., Ha, S. M., Lim, J. M., Kwon, S. J. & Chun, J. A large-scale evaluation of algorithms to calculate average nucleotide identity. *Antonie van Leeuwenhoek* **110**, 1281-1286 (2017).
